# *Ralstonia solanacearum* and *Phytophthora parasitica* promote the infection of each other by disturbing host defense response together

**DOI:** 10.3389/fmicb.2025.1581082

**Published:** 2025-05-09

**Authors:** Xi Liu, Hancheng Wang, Mengru Wang, Yiting Li, Liuti Cai, Junliang Yin

**Affiliations:** ^1^Engineering Research Center of Ecology and Agricultural Use of Wetland, Ministry of Education/MARA Key Laboratory of Sustainable Crop Production in the Middle Reaches of the Yangtze River (Co-construction by Ministry and Province)/Forewarning and Management of Agricultural and Forestry Pests, Hubei Engineering Technology Center/College of Agriculture, Yangtze University, Jingzhou, Hubei, China; ^2^Guizhou Provincial Academician Workstation of Microbiology and Health, Guizhou Academy of Tobacco Science, Guiyang, China

**Keywords:** reactive oxygen species, histochemical staining, *pathogenesis-related* genes, enzyme activity, multi-infection

## Abstract

Multiple infections (multi-infection), either sequential or simultaneous, affecting a single plant or crop are now considered common in plant disease epidemics. The consequences of multi-infection have been studied from the aspects of pathogen virulence, accumulation, transmission, and epidemics, as well as genetic diversity, population structure, and evolutionary trajectory. However, the dynamic changes of host defense response during multi-infection are much unclear. In this study, *Rs* (*Ralstonia solanacearum*) and *Pp* (*Phytophthora parasitica*) were used to simulate the multi-infection and uncover the defense response changes of tobacco. Results showed that the lesion diameter of *Rs*+*Pp* was 350% higher than that of *Pp* and 54.2% higher than that of *Rs*, indicating that co-infection with *Rs* and *Pp* makes tobacco more susceptible to disease. Further analysis showed that co-infection could increase the contents of Aseorbate peroxidase (APX) and Peroxidase (POD), thus lead to the excessive accumulation of Reactive oxygen species (ROS). Meanwhile, most *pathogenesis*-*related* (*PR*) genes were down-regulated, revealing that the immune defense response was disturbed by co-infection and resulted in susceptibility. Our study preliminarily reveals the underlying ways that *Rs* and *Pp* co-infection suppress the host defense response, which will provide a theoretical basis for scientific, reasonable and effective tobacco disease management.

## 1 Introduction

For a long time, diseases in both animals and plants have been thought to be the result of interaction between an individual pathogen and one host (Dutt et al., 2021). Recent years, with the advance of molecular biological technologies, multiple infection (multi-infection) has been shown to have a high prevalence (Tollenaere et al., 2016). Multiple infections that affect a single plant or crop simultaneously or sequentially are now considered common in plant disease epidemics (Dutt et al., 2021). Plants in both natural and agro-ecosystems can encounter a myriad of pathogenic microbes during the growing season (Tollenaere et al., 2016). Pathogen strains entering a host will not only encounter the host’s defenses but also interact directly, indirectly, and/or simultaneously with several other microbial species or genotypes within the plant phytobiomass (Pedersen and Fenton, 2007).

Under multi-infection, these host-mediated or direct interactions can change virulence, within-host pathogen accumulation, and transmission (Alizon, 2013; Gil-Salas et al., 2012). Multi-infection may promote the evolutionary trajectories of pathogen populations by influencing virulence evolution and the generation and maintenance of genetic diversity of pathogen populations. This diversity can fundamentally alter the ability of parasite strains to establish and grow on its host; and dynamics under multi-infection have been considered to be the main driving force for pathogen evolution (Syller, 2012). Such individual-plant-level effects also works at the population level, with multi-infection rendering epidemics more devastating (Syller, 2012). The interactions between or among multi-infection pathogens may be synergistic (i.e., the presence of one parasite may promote the infection of other parasites), or antagonistic (i.e., the presence of one parasite may inhibit the infection of other parasites) (Dutt et al., 2021). Pathogens may infect multiple plants through synergism, which might also explain why certain pathogens are more likely to occur than others in a given environment or plant host.

Tobacco (*Nicotiana tabacum* L.) is one of the world’s most crucial cash crops (Liu X. et al., 2022). Globally, about 33 million people are involved in the tobacco cultivation, product manufacture, retailing and distribution (Warner, 2000). Meanwhile, the global tobacco industry earns an estimated at 2 trillion dollars annually (Martins-Da-Silva et al., 2022). Unfortunately, tobacco is attacked by a variety of fungal and bacterial pathogens throughout the growing season, such as *Phytophthora parasitica*, *Ralstonia solanacearum*, *Fusarium* spp. etc. (Jin et al., 2011; Lamondia, 2015). *Phytophthora parasitica*, the cause agent of tobacco black shank, is an oomycete plant pathogen with broad-range of host plants. It is one of the most notorious tobacco pathogens and causes huge economic losses worldwide (Wang X. et al., 2022; Yu et al., 2024). Tobacco bacterial wilt disease, caused by the *Ralstonia solanacearum*, poses a serious threat to the global flue-cured tobacco crop process (Ahmed et al., 2022; Xiao et al., 2024). Both are soil-borne disease and seriously constrain the safety production and sustainable development of tobacco industry.

At present, the mechanisms behind tobacco defense responses during multiple infections are largely unknown. In this study, with the goal of exploring the interaction between pathogens during tobacco disease development, *Rs* (*Ralstonia solanacearum*) and *Pp* (*Phytophthora parasitica*) were used to simulate the multi-infection. The accumulation of reactive oxygen species, ROS enzyme activity and the expression of *pathogenesis-related* genes were measured. The experimental results revealed the changes of the tobacco defense response. Our results will lay the foundation for rational, scientific and effective prevention and control of tobacco disease.

## 2 Materials and methods

### 2.1 Pathogen culturing

*Rs* (*Ralstonia solanacearum* strain GY1907, provided by Guizhou Academy of Tobacco Science) was cultured on CPG medium (Peptone: 10 g; glucose: 5 g; casamino acid: 1 g; agar: 20 g; distilled water: to 1 L) with 5% TTC (2,3,5-triphenyl-2H-tetrazolium chloride) at 28°C (Cao et al., 2018). After 30 h, *Rs* was cultured in NB liquid medium (Nutrient broth: 18 g; distilled water: to 1 L) until OD_600_ reached 1.0 (28°C, 150 rpm), then diluted with NB medium to OD_600_ = 0.3 (Li et al., 2021). *Pp* (*Phytophthora parasitica* isolate GZMT20, provided by Guizhou Academy of Tobacco Science) was cultured on 5% carrot juice agar medium (Carrot juice: 50 mL; *β*-sitosterol: 0.02 g; CaCO_3_: 0.1 g; distilled water: to 1 L) at 25°C for 3 d in dark (Sullivan et al., 2005).

### 2.2 Tobacco growth and inoculation

Tobacco seeds (cultivar ‘Yunyan 87’, moderately resistant to two pathogens, supplied by Guizhou Academy of Tobacco Science, Guiyang, China) were sterilized by soaking in 1% sodium hypochlorite (NaClO) for 2 min, rinsed by distilled water for three times, then planted in soil and cultured in greenhouse (8 h darkness/16 h light, 25°C, humidity above 50%) (Wang H. et al., 2022). When tobacco plants reached the eight-leaf stage, the 4th to 6th fully expanded leaves from each plant were excised and subjected to four experimental groups: (1) *Rs*, 1 mL of *Rs* suspension was injected into the tobacco leaves according to the method of Wang h. et al. (2015); (2) *Pp*, 6 mm pieces of *Pp* mycelium was attached onto tobacco leaves (Wang H. et al., 2022; Wang M. et al., 2015); (3) *Rs*+*Pp* co-infection, *Rs* suspension was injected and *Pp* mycelium was attached onto the same leaf area; (4) CK treatment, distilled water was injected to tobacco leaves. Then petioles were wrapped with wet cotton to keep moist. Inoculated plants were cultured at 30°C and 16 h light/8 h darkness. Three independent biological replicates were performed, with each replicate consisting of at least 10 leaves. Two days later, the lesion diameter of the leaf yellowing lesions was measured using a vernier caliper and visualized using a handheld long-wavelength UV lamp (Blak-Ray B-100AP, Ultraviolet Products) and photographs were taken using a Canon digital Camera EOS 80D (Japan) (Xiao et al., 2022).

### 2.3 Histochemical staining analysis

To investigate the survival of tobacco cells under different treatments, the inoculated leaves were stained with trypan blue. The leaves were soaked in trypan blue solution (10 g trypan blue powder; 10 g phenol; 10 mL glycerol; 10 mL lactic acid; 10 mL sterile water), boiled in a boiling water bath for 3 min, cooled for 5 min and then boiled for another 2 min. After cooling to room temperature, the leaves were immersed in destaining solution and decolorized for more than 5 h. Histochemical detection of hydrogen peroxide (H_2_O_2_) and super oxide (O^2−^) was determined as described by Zhu et al. (2020) with minor modifications. Briefly, the leaves of different treatments were soaked with 10 mM Tris-acetate (pH 5.0) containing 1 mg/mL DAB (3,3′-diaminobenzidine, MACKLIN, ShangHai, China) for 2 days, vacuum-infiltrated for 5 min and cultured without light at room temperature for 4 h. Leaves were pictured until appearance of brown spots, where represented the reaction of DAB with H_2_O_2_. Moreover, in order to detect the accumulation of O^2−^, leaves were immersed in 10 mM K-phosphate buffer (pH 6.4) containing 0.1% solution of NBT (nitro blue tetrazolium, MACKLIN, ShangHai, China), and were vacuum-infiltrated for 10 min and pictured until appearance of dark spots. Bleach the leaves by soaking them in boiling ethanol (95%, v/v) for 5–10 min to visualize spots.

### 2.4 Enzyme assays

The leaves were collected after 12-, 24-, 36-, and 48-h of treatments, respectively, and used to determine the enzyme activity. Activities of oxidative stress responsive enzymes, including CAT (Catalase), POD (Peroxidase), APX (Aseorbate peroxidase), GSH-Px (Glutathione peroxide), GST (Glutathione S-transferase), DHAR (Dehydroascorbate reductase), GR (Gluathione reductase), and GSH (Glutathione), were detected using kits following the instruction of manufacturer (Suzhou Grace Biotechnology Co., Ltd., Jiangsu, China) (Han et al., 2024). The experiment was performed in three independent replicates with five leaves.

### 2.5 Expression analysis of *pathogenesis*-*related* genes

The 12-, 24-, 36-, and 48-h leaf samples of *Rs*, *Pp*, *Rs*+*Pp* and CK treatments were collected and immediately submerged in liquid nitrogen, then stored at-80°C until further use. Total RNA from leaf samples was extracted with TRizol reagent (GenStar, Beijing, China). The RNA was reverse-transcribed into cDNA using HiScript II 1st Strand cDNA Synthesis Kit (+gDNA wiper) (Vazyme, Nanjing, China). The cDNA was then diluted with RNase-free water to 100 ng/μL. The qRT-PCR was performed on a CFX 96 Real-Time PCR system (Bio Rad, Hercules, CA, United States) using ChamQ SYBR qPCR Master Mix (Vazyme, Nanjing, China) following manufacturer’s instruction (Li et al., 2022). The 20 μL qRT-PCR reaction system consisted of 10 μL of 2 × ChamQ SYBR qPCR Master Mix, 2 μL diluted cDNA, 0.4 μL forward and reverse primers each and 7.2 μL ddH_2_O. The protocol was carried out as following: step 1: pre-denaturation at 95°C for 30 s; step 2: denaturation at 95°C for 5 s; and step 3: primer annealing/extension and collection of fluorescence signal at 58°C for 30 s. The next 40 cycles started from step 2 (Yin et al., 2023). *β-Actin*, which was expressed stably under various conditions, was used as the reference gene for qRT-PCR analysis (Li et al., 2022). The specific primers used in this study were shown in Table 1. Three biological replications were used for each treatment and three technical replicates were used for each cDNA. Relative expression levels were determined using the 2^−ΔΔCt^ method (Yin et al., 2018).

**Table 1:** Sets of primers used for qRT-PCR analyzing the induced patterns of immune defense marker genes.

Name	Sequence (5′–3′)	Pathway	References
*β-Actin*/F	GCTCTCCAACAACATTGCCAAC		Li et al. (2022)
*β-Actin*/R	GCTTCTGCCTGTCACATACGC		
*NbWRKY7*/F	CACAAGGGTACAAACAACACAG	PTI, response to flg22	Wang H. et al. (2019)
*NbWRKY7*/R	GGTTGCATTTGGTTCATGTAAG		
*NbACRE31*/F	AATTCGGCCATCGTGATCTTGGTC	PTI, response to Pep-13	Wang H. et al. (2019)
*NbACRE31*/R	GAGAAACTGGGATTGCCTGAAGGA		
*NbPR3*/F	CAATGCCTTTATCAATGCTG	Jasmonate-dependent defenses	Yin et al. (2020)
*NbPR3*/R	AGTAGTCACCTGGGCTACCT		
*NbPDF1.2*/F	AACTTGTGAGTCCCAGAG	JA/ET-responsive gene	Han et al. (2024)
*NbPDF1.2*/R	GGATACCTTTCTACCACC		
*NbPR4*/F	GATGCTTGAGGGTGACGA	Marker genes of jasmonic acid (JA)-dependent immunity	Nie et al. (2018)
*NbPR4*/R	ATAGCCCACTCCATTTGT		
*NbPR1a*/F	CGTTGAGATGTGGGTCAATG	SAR, response to SA	Lee et al. (2018)
*NbPR1a*/R	CCTAGCACATCCAACACGAA		
*NbPR2*/F	AGGTGTTTGCTATGGAATGC	SAR, response to SA	Lee et al. (2018)
*NbPR2*/R	TCTGTACCCACCATCTTGC		
*NbRbOhA*/F	GACTCGTTCCAGCGCTCATA	H_2_O_2_ accumulation	Yin et al. (2020)
*NbRbOhA*/R	TGTGCGAAATCGGAACGGTA		

### 2.6 Statistical analysis

All experiments were performed with at least three independent biological replicates. Data are presented as mean ± SEM (standard error of the mean). Statistical analyses were conducted using GraphPad Prism 8.0.1 (GraphPad Software, United States). Two-way ANOVA with Tukey’s multiple comparisons test was applied to evaluate the effects of treatments and time points, as well as their interaction. Significant differences (*p* < 0.05) are indicated by different lowercase letters, while shared letters denote no statistically significant difference (*p* > 0.05) between groups (Xi et al., 2022).

## 3 Results

### 3.1 *Rs* and *Pp* mutually promote the colonization

**Figure 1:**
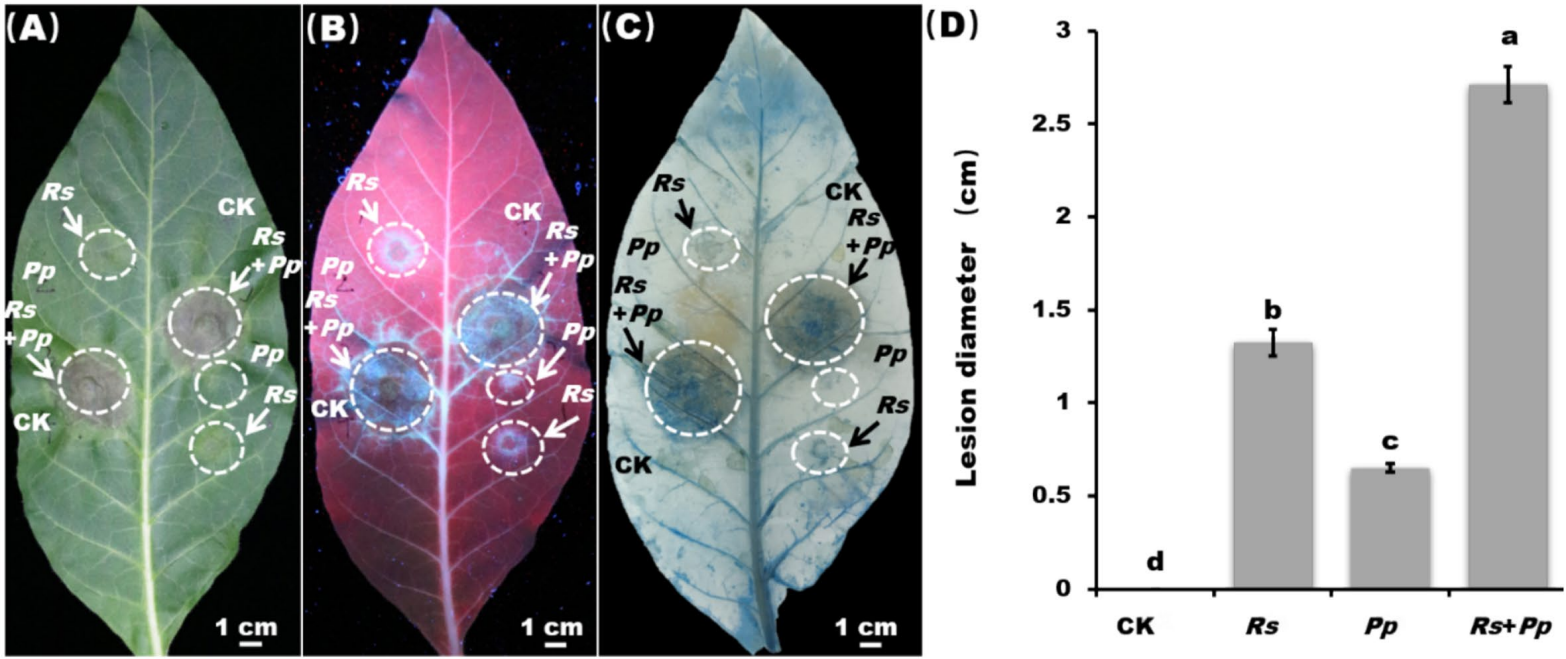
Disease lesion and statistic. Observation of inoculation leaf under **(A)** white light, **(B)** ultraviolet light, and **(C)** trypan blue staining. *Rs*, *Pp*, *Rs*+*Pp*, and CK represent *Ralstonia solanacearum*, *Phytophthora parasitica*, *Rs*+*Pp* co-infection and control. White arrows and circles are the infection area. Bar = 1 cm. **(D)** Statistical analysis of disease lesions. Different letters indicated the significant level at *p* < 0.05.

As shown in Figure 1, the detached leaves grew well under CK treatment, and there were no disease spots. Comparing to CK, the lesion diameter of *Pp* was 0.6 cm and the diameter of *Rs* infection area reached 1.4 cm. Significantly, the *Rs*+*Pp* co-infection was the most serious, with the lesion diameter of 2.7 cm, which was 350% higher than that of *Pp* and 54.2% higher than that of *Rs* (Figures 1A,B,D). Trypan blue staining results showed that the number of dead cells increased significantly when *Rs*+*Pp* infected tobacco simultaneously (Figure 1C). These results showed that the simultaneous infection of *Rs* and *Pp* jointly promoted the disease development.

### 3.2 Activities of enzymes involving in reactive oxygen species

**Figure 2:**
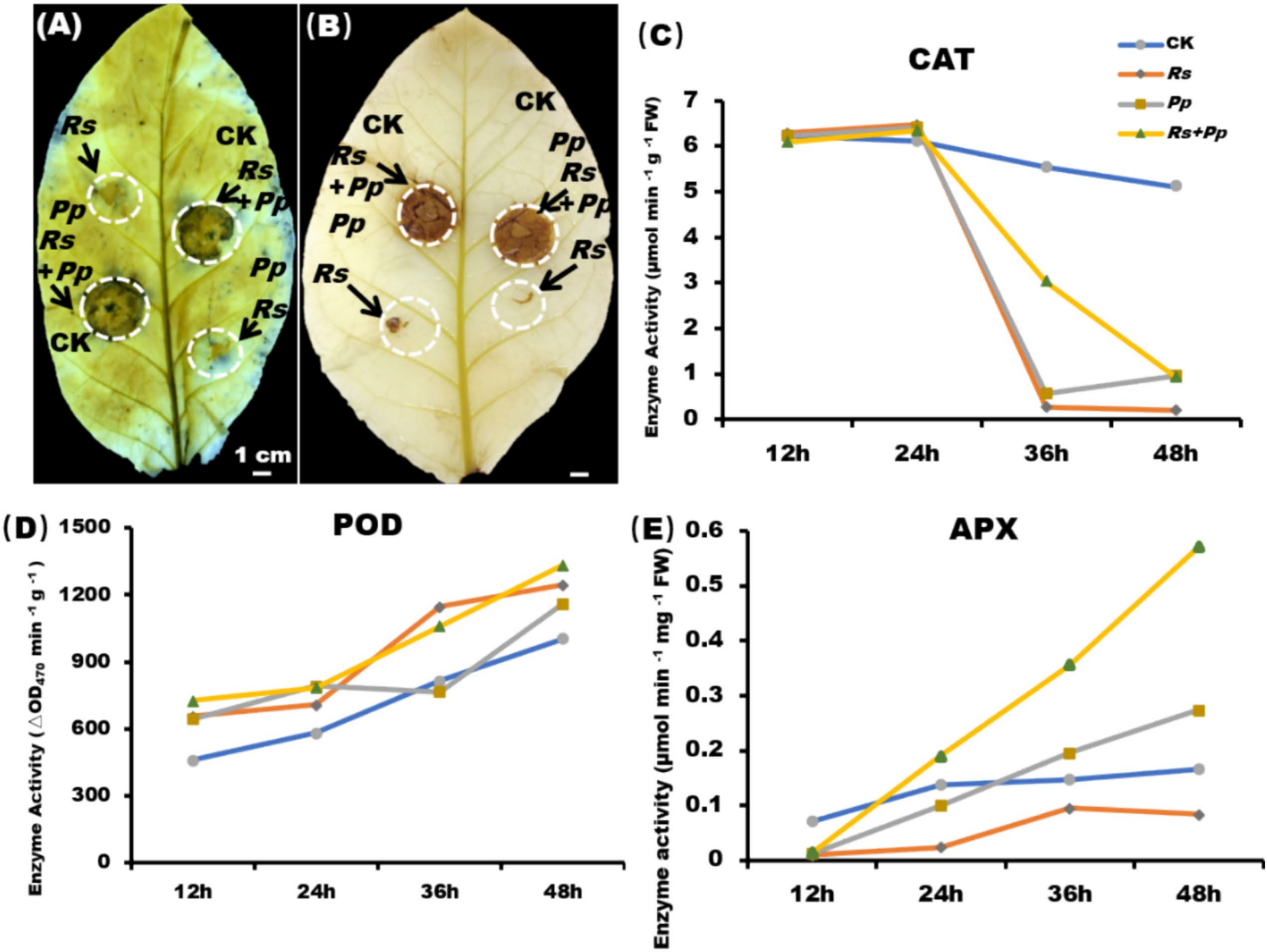
ROS burst and enzyme activity. **(A)** NBT and **(B)** DAB staining of tobacco leaf areas inoculated with *Rs*, *Pp*, *Rs*+*Pp*, and CK. The arrows indicated the **(A)** O^*2*−^ and **(B)** H_2_O_2_ accumulation area. Enzyme activities of **(C)** CAT, **(D)** POD, and **(E)** APX in corresponding leaf areas after 12-, 24-, 36-, and 48-h’ treatment. Different letters indicated the significant level at *p* < 0.05.

The NBT staining indicated that co-infection enhances the accumulation of O^2−^ (Figure 2A), and DAB staining indicated that co-infection increases the accumulation of H_2_O_2_ (Figure 2B). As shown in Figure 2, after 2 days of inoculation, the levels of H_2_O_2_ and O^2−^were significantly increased in the co-infection of *Rs* and *Pp* comparing to their individual infections. Furthermore, it was observed that the levels of H_2_O_2_ and O^2−^ were higher in *Rs*-infected samples alone than in *Pp*-infected samples alone.

Catalase catalyzes the decomposition of hydrogen peroxide into oxygen and water, and its activities largely reflect the capability of plants to scavenge ROS (Liu H. et al., 2022). At 12 h, there was no significant difference in CAT activity among the four treatments. Compared with CK treatment, CAT activity increased slightly in the other three treatments from 12 to 24 h, and significant decreased from 24 to 48 h (Figure 2C).

Peroxidase (POD) utilizes H_2_O_2_ to catalyze oxidation–reduction reactions, oxidizing toxic substances and thereby protecting cells (Yan et al., 2021). POD activity was up-regulated gradually from 12 to 48 h. After 48 h of treatment, the POD enzyme activity of *Rs*, *Pp*, *Rs*+*Pp* co-infection treatments increased by 1.24-, 1.16-, and 1.33-fold, respectively, as compared to that of the CK (Figure 2D). These results indicated that inoculation of pathogens could increase POD activity of tobacco, and the enzyme activity increased the most when *Rs*+*Pp* was co-infected.

Ascorbate peroxidase (APX) plays a crucial role in the detoxification of H_2_O_2_ within chloroplasts. APX activity of four treatments was up-regulated gradually from 12 to 48 h. After 48 h of treatment, compared with CK, *Rs* and *Pp* treatments, the APX activity of *Rs*+*Pp* co-infection was increased 3.43-, 6.86-, and 2.09-fold, respectively (Figure 2E).

### 3.3 Co-infection increases ROS enzyme activity

**Figure 3:**
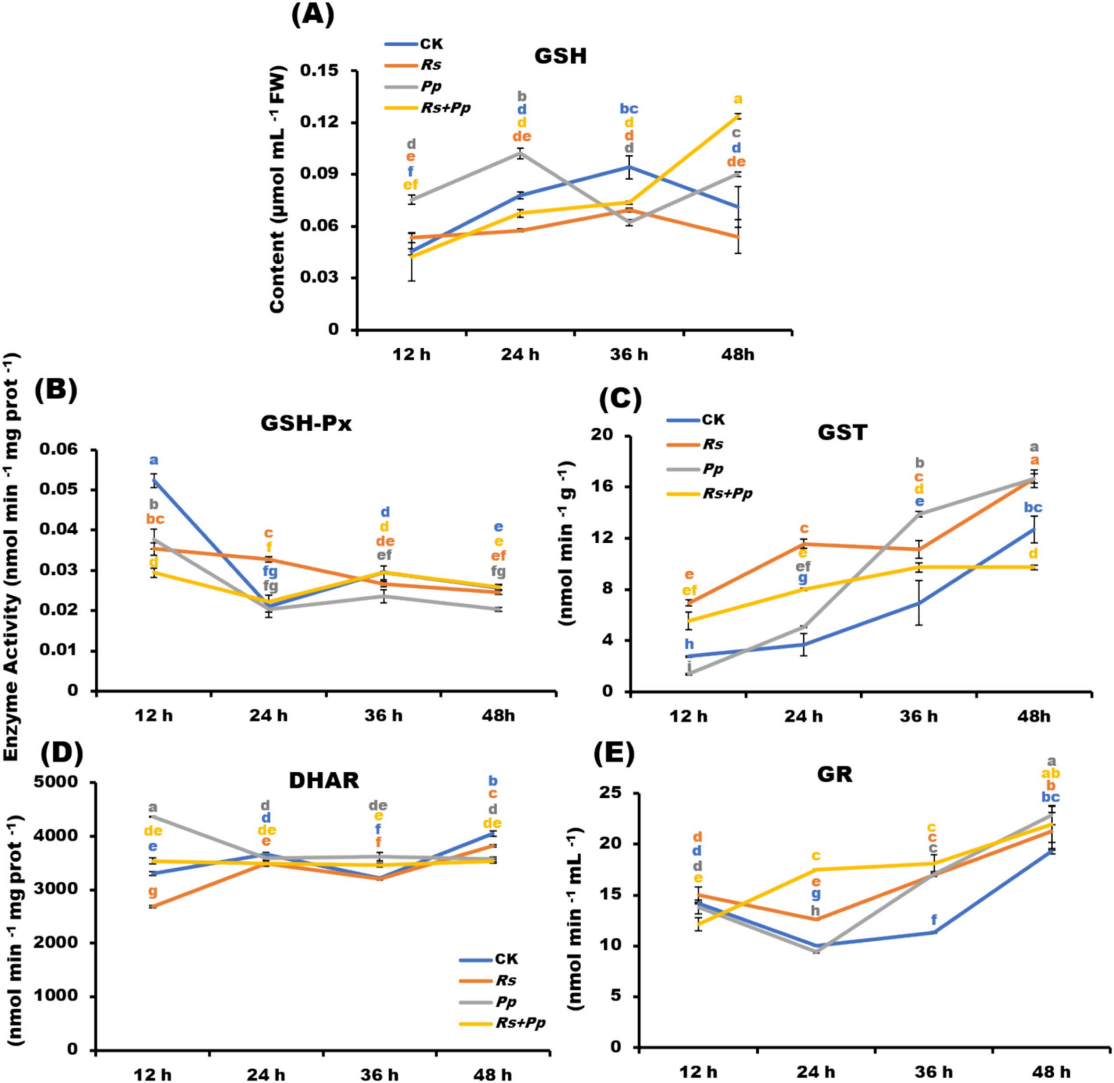
Dynamic changes of **(A)** GSH-Px, **(B)** GST, **(C)** GR, **(D)** DHAR, and **(E)** GSH enzyme activities in *Rs*, *Pp*, *Rs*+*Pp*, and CK treated leaf areas at 12, 24, 36, and 48 h. Different letters indicated the significant level at *p* < 0.05.

Glutathione (Wang X. et al., 2022) plays an important role in amino acid transmembrane transport, protein sulfhydryl protection and antioxidant, which is the most important antioxidant sulfhydryl substance in cells. GSH enzyme activity of four treatments exhibited a fluctuating upward trend. After 48 h of treatment, compared with CK, *Rs* and *Pp* treatments, the GSH activity of *Rs*+*Pp* co-infection was increased 1.74-, 2.3-, and 1.37-fold, respectively (Figure 3A).

Glutathione peroxide (GSH-Px) is widely available, which can promote the decomposition of H_2_O_2_ and protect the structure and function of cell membrane from being interfered with and destroyed by oxides. The GSH-Px enzyme activity of four treatments showed a fluctuating downward trend from 12 to 48 h. After 48 h of treatment, there was little difference between the four treatments (Figure 3B).

Glutathione S-transferase (GST) acts as part of a cellular defense mechanism to remove potentially toxic compounds, including those produced by oxidative stress. GST enzyme activity of four treatments stepped growth from 12 to 48 h. After 48 h of treatment, the GSH activity of *Rs*+*Pp* co-infection increased least (Figure 3C).

Dehydroascorbate reductase is an important antioxidant enzyme in plants and a key enzyme that catalyzes the reduction of dehydroascorbate (Novelli et al., 2009) by GSH (glutathione) to regenerate ascorbic acid (AsA) and oxidized glutathione (GSSG). It plays an important role in maintaining the normal metabolism level of ascorbic acid in plants and protecting cell components against oxidative damage. DHAR activity of four treatments had small changes from 12 to 48 h, the co-infection tended to be stable, especially. The enzyme activity of *Pp* infection down-regulated from 12 to 24 h, and then tended to be stable. The activity of *Rs* infection and CK treatment was up-regulated from 12 to 24 h, down-regulated from 24 to 36 h, and up-regulated from 36 to 48 h (Figure 3D).

Gluathione reductase (GR) catalyzes the oxidation of oxidized glutathione (GSSG) to reduce glutathione (Wang X. et al., 2022). The higher the GSH/GSSG ratio is, the more reactive oxygen species. The GR enzyme activity of *Pp*, *Rs*, and CK treatments was down-regulated from 12 to 24 h, but the activity of co-infection was up-regulated significantly. The activity of four treatments was up-regulated steadily from 24 to 48 h (Figure 3E).

### 3.4 Co-infection affected the expression of pathogenesis-related genes

Two PTI related genes (*NbWRKY7* and *NbACRE31*), three JA-dependent immunity genes (*NbPR3*, *NbPDF1.2*, and *NbPR4*), two SA response genes (*NbPR1a* and *NbPR2*), and one respiratory burst oxidase homologs gene (*NbRbOhA*) were selected to do the expression analysis in these treatment plants.

**Figure 4:**
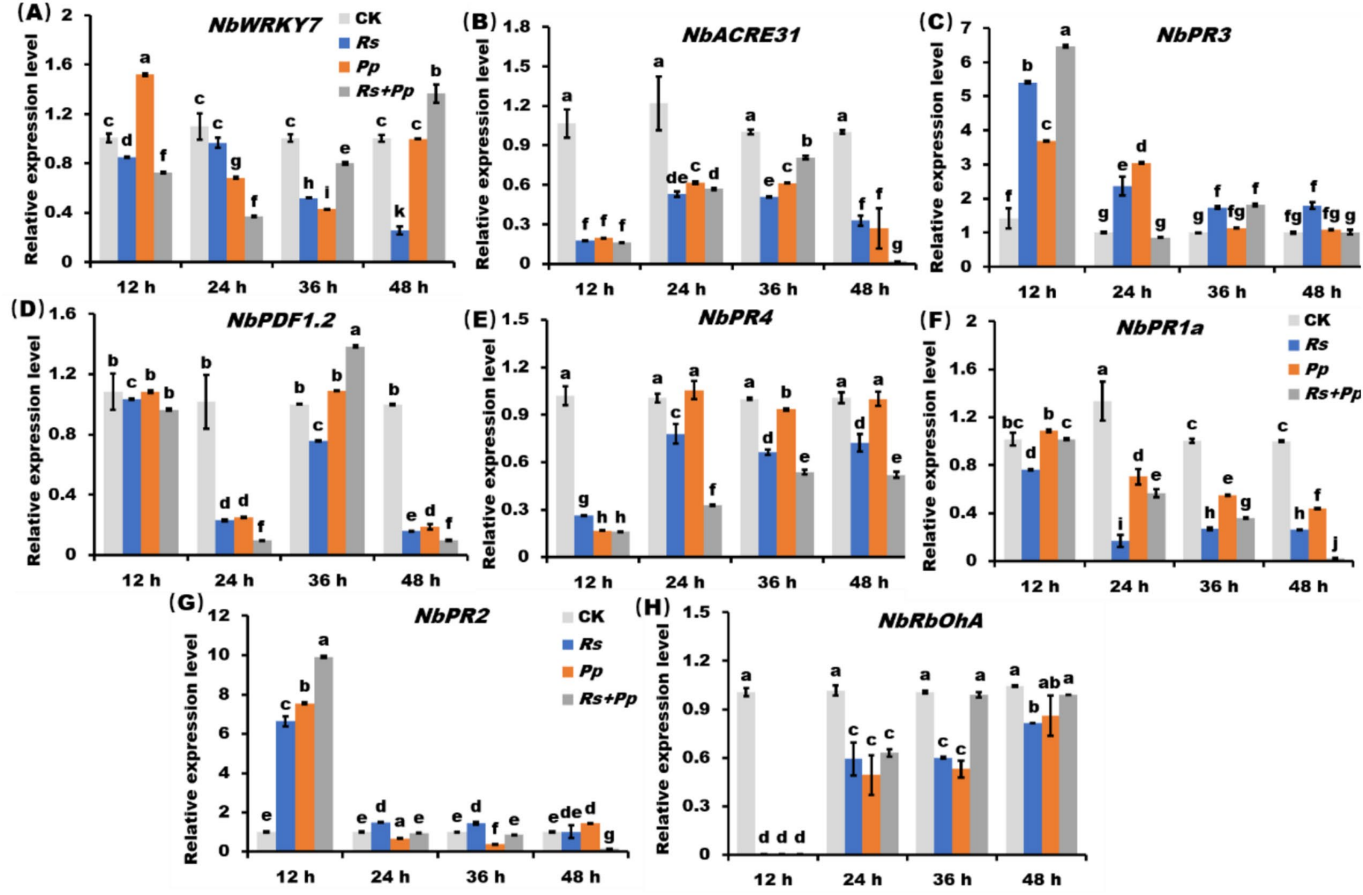
The RT-qPCR analysis of *PR* genes. Relative expression level of **(A)** *NbWRKY7*, **(B)** *NbACRE31*, **(C)** *NbPR3*, **(D)** *NbPDF1.2*, **(E)** *NbPR4*, **(F)** *NbPR1a*, **(G)** *NbPR2*, **(H)** *NbRbOhA* in *Rs*, *Pp*, *Rs*+*Pp*, and CK treated leaf areas at 12, 24, 36, and 48 h. Different letters indicated the significant level at *p* < 0.05.

Compared to infection alone, the *NbWRKY7* gene was significant up-regulated and the *NbACRE31* gene was down-regulated in co-infection of *Rs* and *Pp* within 48 h (Figures 4A,B). The gene expression level of *NbPR3* was up-regulated after 12 h of inoculation with *Rs*, *Pp*, *Rs*+*Pp*; but then steadily declined between 12 and 48 h. Among them, the gene expression of co-infection fluctuated the most (Figure 4C). Compared to CK, the *NbPDF1.2* gene expression showed a fluctuating decrease from 12 to 48 h. The co-infection of *Rs* and *Pp* decreased the least (Figure 4D). Compared to CK, the gene expression level of *NbPR4* gene was significantly down-regulated within 12 h, but went up slightly from 12 to 48 h. (Figure 4E). The gene expression level of *NbPR1a* and *NbPR2* gene was down-regulated gradually within 48 h. The expression level of coinfection down-regulated the least (Figures 4F,G). The *NbRbOhA* gene expression of inoculation with *Rs*, *Pp* and *Rs*+*Pp* tends to zero at 12 h. From 12 to 48 h, its expression increased dramatically, but the gene expression was still down-regulated compared with CK (Figure 4H). These results demonstrated that co-infection of *Rs* and *Pp* could increase the expression of *NbRbOhA* and *NbWRKY7* genes and decrease the expression of *NbACRE31*, *NbPR3*, *NbPDF1.2*, *NbPR4*, *NbPR1a*, *NbPR2*.

## 4 Discussion

Tobacco black shank and bacterial wilt, caused by *Rs* (*Ralstonia solanacearum*) and *Pp* (*Phytophthora parasitica*) respectively, were among the most notorious pathogens of tobacco and resulted in huge economic losses worldwide (Zhang et al., 2019). In the field, *Rs* and *Pp* usually co-infect tobacco, however the dynamic changes behind tobacco defense responses during multiple infections are largely unknown, which limited our ability to reasonable and effective management tobacco diseases.

In this study, *Rs* and *Pp* were employed to simulate multi-pathogen infection, and the alterations in tobacco defense responses were systematically analyzed. The research results showed that *Rs*+*Pp* co-infection caused more serious diseases compared with single infection by *Pp* or *Rs*, as the lesion diameter of *Rs*+*Pp* co-infection was 350% higher than that of *Pp* and 54.2% higher than that of *Rs*; meanwhile, more intense symptoms under co-infection have frequently been observed (Figures 1A–D). To explore this phenomenon, the effects of *Rs* and *Pp* co-infection on disease severity and host anti-infective response were investigated.

*Pp* is a hemibiotrophic pathogen that induces host death at the later stage of infection (Wang X. et al., 2022; Yu et al., 2024). The experimental results showed that the lesion area of *Pp* was small at 48 days of infection, while co-infection was larger, indicating that co-infection promoted the development of the disease. The experimental results also found that the co-infection of *Rs* and *Pp* will promote the production and accumulation of O^2−^ and H_2_O_2_ (Figures 2A,B). When exposed to various environmental stresses such as pathogen infection, high temperature, drought and salt stress, plant cells will be subjected to oxidative stress. These environmental stresses can interfere with the redox balance within the plant, resulting in excessive production of reactive oxygen species (Smith et al., 2007). ROS including hydrogen peroxide (H_2_O_2_), superoxide anion (O^2−^), and hydroxyl radical (OH^−^), possess high oxidative potentials that can induce oxidative damage in living organisms within their cells. Oxidative damage may lead to the damage of biomolecules (such as proteins, lipids, DNA, etc.) in plant cells, thus affecting the structure and function of cells. This can lead to cell inactivation, apoptosis and even death (Tripathi et al., 2023). Therefore, plants have evolved a series of antioxidant systems and defense mechanisms to balance the production and removal of ROS and maintain cell homeostasis superoxide anion (Guo and Cheng, 2022). POD, CAT, and APX serve as important components of antioxidant defense mechanism. Due to its high substrate affinity for H₂O₂ and rapid response to localized H₂O₂ fluctuations, APX exhibits higher enzymatic activity than CAT and POD (Mittler et al., 2004). In this study, POD (Figure 2D) and APX (Figure 2E) enzyme activities increased under co-infection of *Rs* and *Pp*, but the activity of CAT reduced under co-infection (Figure 2C). GSH also plays a crucial role in cellular antioxidant defense (Sharma et al., 2012). The alterations in GSH, GSH-Px, GST, DHAR, and GR levels during co-infection can lead to the accumulation of GSH (Figures 3A–E). However, tobacco did not effectively remove oxidative substances, ROS still accumulate in large quantities in tobacco (Figures 2A,B). Sharma et al. (2012) proved that when cells were in a state of high ROS concentration, oxidative damage and eventual death of cells would be caused.

The JA, SA, PTI pathways, and Active oxygen species (Smith et al., 2007) are central components of the defense reactions of plants against pathogens (Jiao et al., 2022; Wang J. et al., 2019). In the course of pathogen infection, PTI pathway is usually activated first, while JA pathway and SA pathway play an important role in the subsequent defense process (Li et al., 2015). The PTI pathway is activated by PAMPs (pathogen-associated molecular patterns) from pathogens leading to a series of defensive responses, including the production of antimicrobial proteins, the release of reactive oxygen species, and the solidification of cell walls, enhancing resistance to pathogens (Guo and Cheng, 2022). Zhang et al. (2009) found that *NbRbOhA*—silenced *N*. *benthamiana* leaves induced impaired H_2_O_2_ production, indicating that this gene is a major contributor to excitor-induced ROS production. In this study, experimental results found *NbRbOhA* and *NbWRKY7* gene in co-infection were up-regulated compared to infection of *Rs* and *Pp* alone at 36 and 48 h (Figures 4A,H). This result further proves that co-infection leads to the accumulation of ROS in tobacco. The expressions of *NbPR2* and *NbPR3 g*enes were significantly up-regulated at 12 h (Figures 4C,G), *NbPDF1.2* and *NbACRE31* genes were significantly up-regulated at 36 h (Figures 4B,D). Zhang et al. (2009) also found that *PR* gene expression is accompanied by hypersensitive response (HR) of cell, which leads to death. This study showed that co-infection causes tobacco cells to be in a state of high ROS concentration, which may lead to cell dysfunction and even death. Therefore, the basic survival mechanisms of cells may be impaired, including the regulation of *PR* gene expression, making it more vulnerable to pathogens and other external pressures (Sharma et al., 2012). This can lead to the occurrence and spread of disease. One may concern that the *Pp* mainly infects the root and stem base of tobacco, and *Rs* is the causal agent of bacterial wilt, which is normally infects through wounds in roots. In this study, leaves were employed to performance the stimulation of co-infection, and may not exactly reflect the defense changes of host plants. Actually, in numerous studies surrounding *Pp* and/or *Rs*, leaves were employed as experimental organ to investigate the defense response of tobacco plants (Judith et al., 2005; Sun et al., 2017). Therefore, it does make sense for using leaves to study defense response of *Pp* and *Rs* co-infection.

Collectively, as indicated by this study, the pathogenic ability of *Pp* and *Rs* to tobacco will be enhanced when they are co-infected, which suggests that when one disease occurs, we should pay attention to the other disease, and use effective fungicides for both pathogens to control and prevent the disease process of both sides. Meanwhile, it should be noticed that this study is an attempt to preliminarily explore the possible reasons underlying the co-infection, nevertheless there are still lots of unanswered questions. For example, do the *Pp* and *Rs* benefit each other by secret different effectors to manipulate host defense together? Do the changes of issues upon one disease make the easier infection of another pathogen? In the next step, we will carry out in-depth research on these questions.

## 5 Conclusion

This study reveals that the co-infection of *Ralstonia solanacearum* and *Phytophthora parasitica* significantly enhances pathogenicity in tobacco through coordinated regulation of ROS metabolism and interference with plant immune signaling pathways. The synergistic infection not only induces a stronger oxidative burst but also disrupts the antioxidant system, leading to impaired ROS scavenging capacity. Concurrently, the two pathogens collectively suppress the expression of key marker genes in both SA and JA signaling pathways, while selectively activating certain PTI-related genes. This dual strategy, simultaneously exacerbating oxidative damage and suppressing immune responses, ultimately results in more severe lesion expansion and cell death. The findings provide novel insights into the synergistic effects of co-infection in plant-pathogen interactions and offer important implications for developing control strategies against complex diseases.

## Data Availability

The original contributions presented in the study are included in the article/supplementary material, further inquiries can be directed to the corresponding authors.

## References

[ref1] Ahmed W. Yang J. Tan Y. Munir S. Liu Q. Zhang J.. (2022). *Ralstonia solanacearum*, a deadly pathogen: revisiting the bacterial wilt biocontrol practices in tobacco and other Solanaceae. Rhizosphere 21:100479. doi: 10.1016/j.rhisph.2022.100479

[ref2] Alizon S. (2013). Parasite co-transmission and the evolutionary epidemiology of virulence. Evolution 67, 921–933. doi: 10.1111/j.1558-5646.2012.01827.x 23550745

[ref3] Cao Y. Pi H. Chandrangsu P. Li Y. Wang Y. Zhou H.. (2018). Antagonism of two plant-growth promoting *Bacillus velezensis* isolates against *Ralstonia solanacearum* and *Fusarium oxysporum*. Sci. Rep. 8:4360. doi: 10.1038/s41598-018-22782-z, PMID: 29531357 PMC5847583

[ref4] Dutt A. Andrivon D. Le May C. (2021). Multi-infections, competitive interactions, and pathogen coexistence. Plant Pathol. 71, 5–22. doi: 10.1111/ppa.13469, PMID: 40265321

[ref5] Gil-Salas F. M. Peters J. Boonham N. Cuadrado I. M. Janssen D. (2012). Co-infection with cucumber vein yellowing virus and cucurbit yellow stunting disorder virus leading to synergism in cucumber. Plant Pathol. 61, 468–478. doi: 10.1111/j.1365-3059.2011.02545.x

[ref6] Guo J. Cheng Y. L. (2022). Advances in fungal elicitor-triggered plant immunity. Int. J. Mol. Sci. 23:12003. doi: 10.3390/ijms231912003, PMID: 36233304 PMC9569958

[ref7] Han S. Han X. Li Y. Li K. Yin J. Gong S.. (2024). Wheat lesion mimic homology gene TaCAT2 enhances plant resistance to biotic and abiotic stresses. Int. J. Biol. Macromol. 277:134197. doi: 10.1016/j.ijbiomac.2024.134197, PMID: 39069064

[ref8] Jiao L. Bian L. Luo Z. Li Z. Xiu C. Fu N.. (2022). Enhanced volatile emissions and anti-herbivore functions mediated by the synergism between jasmonic acid and salicylic acid pathways in tea plants. Horticult. Res. 9:uhac144. doi: 10.1093/hr/uhac144, PMID: 36101895 PMC9463459

[ref9] Jin F. Ding Y. Wei D. Reddy M. S. Du B. (2011). Genetic diversity and phylogeny of antagonistic Bacteria against *Phytophthora nicotianae* isolated from tobacco *rhizosphere*. Int. J. Mol. Sci. 12, 3055–3071. doi: 10.3390/ijms12053055, PMID: 21686169 PMC3116175

[ref10] Judith S. Hardy S. Engelbert W. (2005). Photosynthesis and carbohydrate metabolism in tobacco leaves during an incompatible interaction with *Phytophthora nicotianae*. Plant Cell Environ. 28, 1421–1435. doi: 10.1111/j.1365-3040.2005.01380.x, PMID: 40265321

[ref11] Lamondia J. A. (2015). Fusarium wilt of tobacco. Crop Prot. 73, 73–77. doi: 10.1016/j.cropro.2015.03.003

[ref12] Lee H. Lee S. Park S. Kleeff P. J. M. V. Schuurink R. C. Ryu C. M. (2018). Transient expression of whitefly effectors in *Nicotiana benthamiana* leaves activates systemic immunity against the leaf pathogen *pseudomonas syringae* and soil-borne pathogen *Ralstonia solanacearum*. Front. Ecol. Evol. 6:90. doi: 10.3389/fevo.2018.00090

[ref13] Li Y. Liu X. Xiao Y. Wen Y. Li K. Ma Z.. (2022). Genome-wide characterization and function analysis uncovered roles of wheat LIMs in responding to adverse stresses and *TaLIM8-4D* function as a susceptible gene. Plant Genome 15:e20246. doi: 10.1002/tpg2.20246, PMID: 35894660 PMC12807053

[ref14] Li S. Pi J. Zhu H. Yang L. Zhang X. Ding W. (2021). Caffeic acid in tobacco root exudate defends tobacco plants from infection by *Ralstonia solanacearum*. Front. Plant Sci. 12:690586. doi: 10.3389/fpls.2021.690586, PMID: 34456935 PMC8387680

[ref15] Li X. Wu J. Yin L. Zhang Y. Qu J. Lu J. (2015). Comparative transcriptome analysis reveals defense-related genes and pathways against downy mildew in *Vitis amurensis* grapevine. Plant Physiol. Biochem. 95, 1–14. doi: 10.1016/j.plaphy.2015.06.016, PMID: 26151858

[ref16] Liu H. Hu H. Tang K. Rehman M. Du G. Huang Y.. (2022). Overexpressing hemp salt stress induced transcription factor genes enhances tobacco salt tolerance. Ind. Crop. Prod. 177:114497. doi: 10.1016/j.indcrop.2021.114497

[ref17] Liu X. Li Y. Wang M. Cao P. Wang H. Yin J. (2022). Stem-end rot caused by *Lasiodiplodia brasiliense* on tobacco in China. Plant Dis. 106:3208. doi: 10.1094/PDIS-03-22-0462-PDN, PMID: 35486604

[ref18] Martins-Da-Silva A. S. Torales J. Becker R. F. V. Moura H. F. Campos M. W. Fidalgo T. M.. (2022). Tobacco growing and tobacco use. Int. Rev. Psychiatry 34, 51–58. doi: 10.1080/09540261.2022.2034602, PMID: 35584014

[ref19] Mittler R. Vanderauwera S. Gollery M. Breusegem V. K. (2004). Reactive oxygen gene network of plants. Trends Plant Sci. 9, 490–498. doi: 10.1016/j.tplants.2004.08.009, PMID: 15465684

[ref20] Nie J. Yin Z. Li Z. Wu Y. Huang L. (2018). A small cysteine-rich protein from two kingdoms of microbes is recognized as a novel pathogen-associated molecular pattern. New Phytol. 222, 995–1011. doi: 10.1111/nph.15631, PMID: 30537041

[ref21] Novelli J. F. Chaudhary K. Canovas J. Benner J. S. Madinger C. L. Kelly P.. (2009). Characterization of the *Caenorhabditis elegans* UDP-galactopyranose mutase homolog glf-1 reveals an essential role for galactofuranose metabolism in nematode surface coat synthesis. Dev. Biol. 335, 340–355. doi: 10.1016/j.ydbio.2009.09.010, PMID: 19751718

[ref22] Pedersen A. B. Fenton A. (2007). Emphasizing the ecology in parasite community ecology. Trends Ecol. Evol. 22, 133–139. doi: 10.1016/j.tree.2006.11.005, PMID: 17137676

[ref23] Sharma P. Jha A. B. Dubey R. S. Pessarakli M. (2012). Reactive oxygen species, oxidative damage, and antioxidative defense mechanism in plants under stressful conditions. J. Bot. 2012, 1–26. doi: 10.1155/2012/217037, PMID: 40102779

[ref24] Smith T. Rana R. S. Missiaen P. Rose K. D. Sahni A. Singh H.. (2007). High bat (*Chiroptera*) diversity in the early Eocene of India. Naturwissenschaften 94, 1003–1009. doi: 10.1007/s00114-007-0280-9, PMID: 17671774

[ref25] Sullivan M. J. Melton T. A. Shew H. D. (2005). Managing the race structure of *Phytophthora parasitica* var. *nicotianae* with cultivar rotation. Plant Dis. 89, 1285–1294. doi: 10.1094/PD-89-1285, PMID: 30791306

[ref26] Sun Y. Li P. Deng M. Shen D. Dai G. Yao N.. (2017). The *Ralstonia solanacearum* effector RipAK suppresses plant hypersensitive response by inhibiting the activity of host catalases. Cell. Microbiol. 19:e12736. doi: 10.1111/cmi.12736, PMID: 28252830

[ref27] Syller J. (2012). Facilitative and antagonistic interactions between plant viruses in mixed infections. Mol. Plant Pathol. 13, 204–216. doi: 10.1111/j.1364-3703.2011.00734.x, PMID: 21726401 PMC6638836

[ref28] Tollenaere C. Susi H. Laine A. L. (2016). Evolutionary and epidemiological implications of multiple infection in plants. Trends Plant Sci. 21, 80–90. doi: 10.1016/j.tplants.2015.10.014, PMID: 26651920

[ref29] Tripathi D. Oldenburg D. J. Bendich A. J. (2023). Oxidative and glycation damage to mitochondrial DNA and plastid DNA during plant development. Antioxidants 12:891. doi: 10.3390/antiox12040891, PMID: 37107266 PMC10135910

[ref30] Wang H. He H. Qi Y. Mclellan H. Tian Z. Birch P. R. J.. (2019). The oomycete microbe-associated molecular pattern Pep-13 triggers SERK3/BAK1-independent plant immunity. Plant Cell Rep. 38, 173–182. doi: 10.1007/s00299-018-2359-5, PMID: 30488097

[ref31] Wang H. Li Y. Li W. Cai L. Meng J. Xia G.. (2022). Morphological and molecular identification of *Fusarium ipomoeae* as the causative agent of leaf spot disease in tobacco from China. Microorganisms 10:1890. doi: 10.3390/microorganisms10101890, PMID: 36296167 PMC9611381

[ref32] Wang J. Liu X. Zhang A. Ren Y. Wu F. Wang G.. (2019). A cyclic nucleotide-gated channel mediates cytoplasmic calcium elevation and disease resistance in rice. Cell Res. 29, 820–831. doi: 10.1038/s41422-019-0219-7, PMID: 31444468 PMC6796908

[ref33] Wang M. Wang H. Huang Y. Wang J. Zhang C. Lu H. (2015). Phenotypic analysis of *Phytophthora parasitica* by using high throughput phenotypic microarray. Acta Microbiol Sin. 55, 1356–1363. doi: 10.13343/j.cnki.wsxb.20150036, PMID: 26939465

[ref34] Wang H. Wang J. Xia H. Huang Y. Wang M. Jia M.. (2015). Sensitivities of *Ralstonia solanacearum* to streptomycin, calcium oxide, mancozeb and synthetic fertilizer. Plant Pathol. J. 14, 13–22. doi: 10.3923/ppj.2015.13.22

[ref35] Wang X. Xie Y. Li Z. Chen Q. Sun J. Han X.. (2022). Honokiol inhibits growth and improves control efficiency against *Phytophthora nicotianae* through disrupting redox homeostasis and energy metabolism. Ind. Crop. Prod. 178:114656. doi: 10.1016/j.indcrop.2022.114656

[ref36] Warner K. E. (2000). The economics of tobacco: myths and realities. Tob. Control. 9, 78–89. doi: 10.1136/tc.9.1.78, PMID: 10691761 PMC1748316

[ref37] Xi K. Xiong S. Li G. Guo C. Zhou J. Ma J.. (2022). Antifungal activity of ginger rhizome extract against *Fusarium solani*. Horticulturae 8:983. doi: 10.3390/horticulturae8110983

[ref38] Xiao Z. Liu Z. Zhang H. Yang A. Cheng L. Liu D.. (2024). Transcriptomics and virus-induced gene silencing identify defense-related genes during *Ralstonia solanacearum* infection in resistant and susceptible tobacco. Genomics 116:110784. doi: 10.1016/j.ygeno.2024.110784, PMID: 38199265

[ref39] Xiao Y. Zhang J. Li Y. Hsiang T. Zhang X. Zhu Y.. (2022). An efficient overexpression method for studying genes in *Ricinus* that transport vectorized agrochemicals. Plant Methods 18:11. doi: 10.1186/s13007-022-00842-w, PMID: 35081982 PMC8793271

[ref40] Yan X. Guan Y. Liu X. Yu J. Lei B. Wang Z.. (2021). *NtCycB2* gene knockout enhances resistance to high salinity stress in *Nicotiana tabacum*. Ind. Crop. Prod. 171:113886. doi: 10.1016/j.indcrop.2021.113886

[ref41] Yin J. Gu B. Huang G. Tian Y. Quan J. Lindqvist-Kreuze H.. (2018). Conserved RXLR effector genes of *Phytophthora infestans* expressed at the early stage of potato infection are suppressive to host defense. Front. Plant Sci. 8:2155. doi: 10.3389/fpls.2017.02155, PMID: 29312401 PMC5742156

[ref42] Yin J. Wang L. Zhao J. Li Y. Huang R. Jiang X.. (2020). Genome-wide characterization of the C_2_H_2_ zinc-finger genes in *Cucumis sativus* andfunctional analyses of four CsZFPs inresponse to stresses. BMC Plant Biol. 20:359. doi: 10.1186/s12870-020-02575-1, PMID: 32727369 PMC7392682

[ref43] Yin X. Yuan Y. Han X. Han S. Li Y. Ma D.. (2023). Genome-wide identification, characterization, and expression profiling of TaDUF668 gene family in *Triticum aestivum*. Agronomy 13:2178. doi: 10.3390/agronomy13082178 PMC10531524 37762550

[ref44] Yu B. Li J. Moussa M. G. Wang W. Song S. Xu Z.. (2024). Molybdenum inhibited the growth of *Phytophthora nicotiana* and improved the resistance of *Nicotiana tabacum* L. against tobacco black shank. Pestic. Biochem. Physiol. 199:105803. doi: 10.1016/j.pestbp.2024.105803, PMID: 38458661

[ref45] Zhang H. Fang Q. Zhang Z. Wang Y. Zheng X. (2009). The role of respiratory burst oxidase homologues in elicitor-induced stomatal closure and hypersensitive response in *Nicotiana benthamiana*. J. Exp. Bot. 60, 3109–3122. doi: 10.1093/jxb/erp146, PMID: 19454596 PMC2718215

[ref46] Zhang Q. Feng R. Zheng Q. Li J. Liu Z. Zhao D.. (2019). Population genetic analysis of *Phytophthora parasitica* from tobacco in Chongqing. Plant Dis. 103, 2599–2605. doi: 10.1094/PDIS-05-18-0879-RE, PMID: 31339441

[ref47] Zhu Y. Jiang X. Zhang J. He Y. Zhu X. Zhou X.. (2020). Silicon confers cucumber resistance to salinity stress through regulation of proline and cytokinins. Plant Physiol. Biochem. 156, 209–220. doi: 10.1016/j.plaphy.2020.09.014, PMID: 32977177

